# Correlation between intravesical prostatic protrusion and ejaculatory dysfunction after tamsulosin treatment in men with BPH

**DOI:** 10.1007/s00345-026-06266-8

**Published:** 2026-02-17

**Authors:** Cem Tuğrul Gezmiş, Nusret Can Çilesiz, Veli Huzeyfe Kartal, Mahmut Kemal Altan, Basri Çakıroğlu, Mustafa Bahadır Can Balcı

**Affiliations:** 1 Department of Urology, University of Health Sciences Türkiye, Taksim Training and Research Hospital, Istanbul, Turkey; 2 Urology Department, Hisar Intercontinental Hospital, Istanbul, Turkey

**Keywords:** Benign prostatic hyperplasia, Ejaculation disorders, Lower urinary tract symptoms, Male sexual health, Tamsulosin, Ultrasonography

## Abstract

**Purpose:**

To evaluate the correlation between prostate morphological measurements and changes in ejaculatory function during short-term tamsulosin therapy in men with benign prostatic hyperplasia (BPH).

**Methods:**

This prospective study included 214 sexually active men with moderate-to-severe lower urinary tract symptoms (LUTS) who received tamsulosin 0.4 mg/day for eight weeks. Patients with prior treatment for BPH or pre-existing sexual dysfunction were excluded. Symptom severity and ejaculatory function were evaluated using the International Prostate Symptom Score (IPSS) and the Male Sexual Health Questionnaire–Ejaculatory Dysfunction Short Form (MSHQ-EjD-SF). Prostate morphological measurements, including overall dimensions and intravesical prostatic protrusion (IPP), were obtained by transabdominal ultrasonography. Correlations between changes in MSHQ-EjD scores (ΔMSHQ-EjD) and clinical or anatomical variables were assessed using Spearman’s rank coefficients.

**Results:**

After eight weeks of treatment, the median IPSS significantly decreased from 18 to 14 (*p* < 0.001) and Qmax increased from 9.2 to 14.0 mL/s (*p* < 0.001). The median MSHQ-EjD-SF score declined from 11 to 7 (*p* < 0.001), while higher ejaculatory bother scores were observed after treatment. ΔMSHQ-EjD was negatively correlated with IPP (ρ = −0.386), prostate length (ρ = −0.250), and age (ρ = −0.334) (all *p* < 0.01). Patients with IPP > 10 mm showed a less pronounced decline in ejaculatory function compared with lower IPP grades (*p* < 0.001).

**Conclusions:**

Tamsulosin improved urinary symptoms but was associated with a decline in ejaculatory function. Prostate morphological characteristics, particularly IPP > 10 mm and longer prostate length, were associated with a less pronounced decline in ejaculatory function. Although these correlations were modest, the findings may help contextualize the relationship between prostate anatomy and tamsulosin-related ejaculatory changes.

## Introduction

Benign prostatic hyperplasia (BPH) is a prevalent condition among aging men, characterized by prostate enlargement that contributes significantly to lower urinary tract symptoms (LUTS) and impairs quality of life. Epidemiological studies have shown that the histological prevalence of BPH increases with age, reaching 50–60% in men in their 60s and up to 80–90% in those over 70 years [1]. This growing burden highlights the need for effective and well-tolerated treatment options.

Alpha-1 adrenoceptor antagonists (α-blockers), such as tamsulosin, doxazosin, and silodosin, are widely accepted as first-line pharmacological therapy for symptomatic BPH because they relax prostatic smooth muscle, thereby improving urinary flow and alleviating LUTS [2–4]. Tamsulosin has been shown to significantly reduce the International Prostate Symptom Score (IPSS) by approximately 6 points and increase the maximum urinary flow rate (Qmax) by about 4 mL/s in a recent meta-analysis [5].

Despite their clinical efficacy, α-blockers are associated with sexual side effects, particularly ejaculatory dysfunction (EjD) [6–8]. Tamsulosin effectively improves lower urinary tract symptoms, while carrying an approximately 4–26% risk of ejaculatory dysfunction, depending on the dose and duration of treatment [9, 10]. A previous meta-analysis demonstrated that EjD was significantly more common with tamsulosin than with placebo and was correlated with greater improvements in IPSS and Qmax [11]. Song et al. further reported that reductions in the Male Sexual Health Questionnaire-Ejaculatory Dysfunction (MSHQ-EjD) score were more frequent among symptomatic responders, particularly in patients with smaller prostates and higher baseline MSHQ scores [12].

This study aimed to evaluate the association between prostate morphological parameters—particularly intravesical prostatic protrusion (IPP)—and changes in ejaculatory function after tamsulosin therapy in men with clinical BPH.

## Materials and methods

This prospective cohort study was conducted at the Department of Urology, Taksim Training and Research Hospital, between March and August 2025, following approval from the institutional ethics committee. Sexually active men who were initiated on tamsulosin 0.4 mg/day for clinically diagnosed BPH were enrolled.

The study included patients presenting with moderate-to-severe LUTS (IPSS > 8) who had no prior medical or surgical treatment for BPH and reported no erectile dysfunction, defined as an International Index of Erectile Function–5 (IIEF-5) score ≥ 22, and preserved ejaculatory function at baseline, defined as an MSHQ-EjD Q1–Q3 score ≥ 8, consistent with previously used descriptive thresholds in the literature [13, 14].

Patients were excluded if they had type 1 or type 2 diabetes mellitus, neurological disorders causing nerve damage (such as spinal cord injury, multiple sclerosis, or Parkinson’s disease), peripheral neuropathies, hyperprolactinemia, prostate cancer or other malignancies, thyroid dysfunction, hypogonadism or other hormonal disorders, use of antidepressants (SSRIs or TCAs) or antipsychotics, major depression or obsessive-compulsive disorder, alcohol or substance dependence, hypertension treated with alpha-blockers, use of beta-blockers or diuretics, chronic kidney or liver failure, or a history of pelvic or urological surgery. Patients with surgical indications for BPH, such as recurrent urinary tract infections, urinary retention requiring catheterization, macroscopic hematuria, bladder stones, upper urinary tract dilatation, or renal failure, were also excluded from the study. Patients who discontinued the medication due to adverse effects were excluded as well.

Baseline evaluations included detailed medical and urological history, digital rectal examination, laboratory analyses (complete urinalysis, urea, creatinine, prostate-specific antigen, and urine culture), uroflowmetry, and ultrasonography (USG). Uroflowmetry was performed with a minimum voided volume of 200 mL at baseline and after 8 weeks of treatment, and the maximum flow rate (Qmax, mL/s) and voided volume were recorded.

### Ultrasonographic measurement protocol

USG assessments were performed using a Toshiba Aplio 300^®^ device equipped with PVT-375 BT convex transducer (frequency range, 1.9–6.0 MHz) transabdominal convex probe(Toshiba Medical Systems, Tokyo, Japan). The prostate volume was estimated by obtaining three linear dimensions. In the transverse plane, the width (D1) was measured as the maximal horizontal distance between the lateral borders of the gland, while the anteroposterior height (D2) was measured from the anterior surface to the posterior capsule in the same section. The longitudinal length (D3) was then obtained in the sagittal view, extending from the prostatic base to the apex. The prostate volume (mL) was calculated using the following formula: prostate volume = 0.52 × width (D1) × height (D2) × length (D3).

IPP was measured in the sagittal plane as the vertical distance (mm) from the bladder neck to the apex of the protruding prostate. IPP was classified as Grade I (< 5 mm), Grade II (5–10 mm), or Grade III (> 10 mm).

### Questionnaire assessment

Symptom severity was evaluated at baseline and after treatment using the validated Turkish version of the IPSS, while ejaculatory function was assessed with the validated Turkish version of the Male Sexual Health Questionnaire–Ejaculatory Dysfunction Short Form (MSHQ-EjD-SF) [13, 15].

The IPSS consists of seven questions scored from 0 to 5, yielding a total score of 0–35, with severity categorized as mild (0–7), moderate (8–19), and severe (20–35), supplemented by a separate quality-of-life question.

The MSHQ-EjD-SF includes four questions: the first three assess ejaculation frequency, strength, and semen volume, each scored from 1 to 5, with higher scores reflecting better function; the fourth question evaluates the degree of bother related to ejaculatory problems, scored from 0 to 5, where higher values indicate greater bother.

### Statistical analysis

Continuous variables were tested for normality using the Shapiro–Wilk test. Depending on the distribution pattern, data were expressed as either mean ± standard deviation (SD) or median (interquartile range, IQR).

Paired comparisons between pre- and post-treatment values were conducted using the Wilcoxon signed-rank test. Changes among groups were evaluated with the Kruskal–Wallis test, followed by Dunn–Bonferroni post-hoc analyses for pairwise group comparisons. The relationship between ΔMSHQ-EjD and continuous clinical or anatomical variables was evaluated using Spearman’s rank correlation coefficients (ρ), reported with corresponding ρ and p values. All statistical tests were two-tailed, and a p value < 0.05 was considered statistically significant.

All statistical analyses were performed using R software (version 4.5.1; R Foundation for Statistical Computing, Vienna, Austria).

## Results

**Fig. 1:**
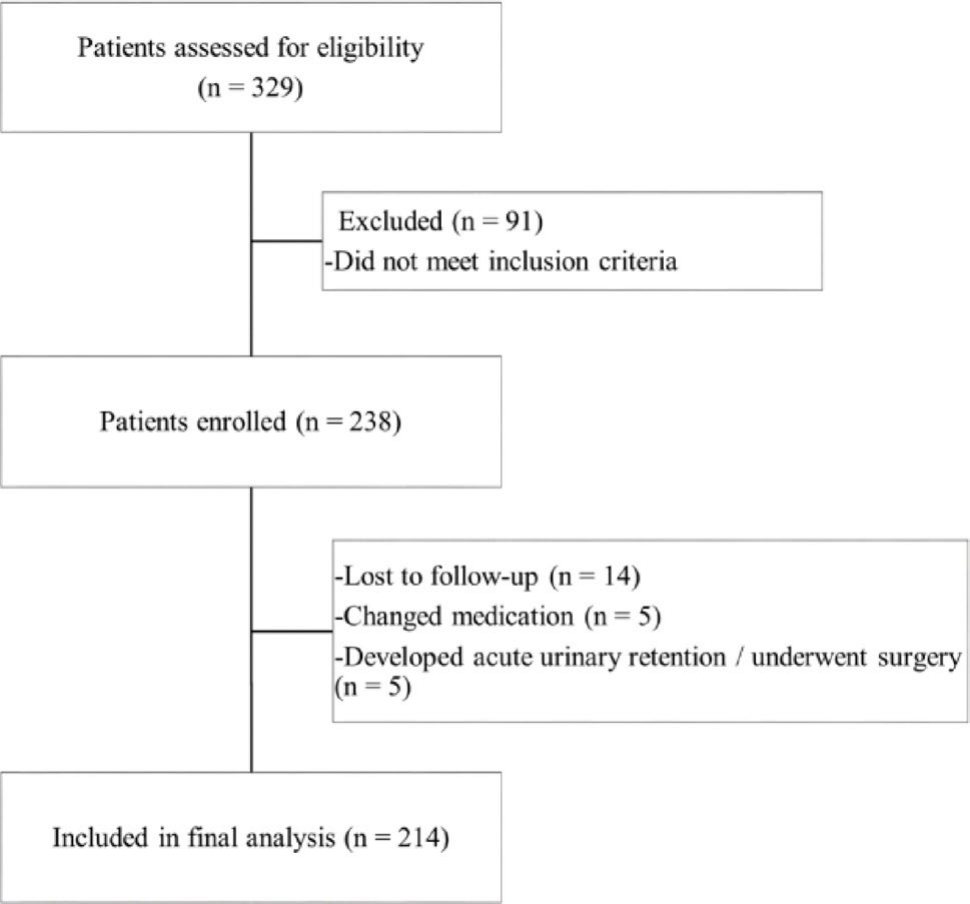
Flow diagram of patient selection and inclusion in the study

Of the 329 patients evaluated for eligibility, 214 successfully completed the study and were included in the final analysis (Fig. 1).

The baseline demographic and clinical characteristics of the study population are summarized in Table 1. The median age was 61 years (IQR, 56–66), and the median BMI was 26.4 kg/m² (IQR, 25.0–28.3). The median PSA level was 1.3 ng/mL (IQR, 0.8–2.75). Among comorbidities, hypertension (26.1%) and coronary artery disease (5.6%) were the most common.

Regarding prostate morphology, the median width (D1), height (D2), and length (D3) were 4.44 cm (IQR, 4.04–4.89), 4.36 cm (IQR, 3.93–4.87), and 4.45 cm (IQR, 3.96–5.29), respectively, with a median prostate volume of 45.0 mL (IQR, 34.0–63.5). According to IPP grading, 34.1% of patients were classified as Grade I (< 5 mm), 36.4% as Grade II (5–10 mm), and 29.4% as Grade III (> 10 mm) (Table 1). Baseline demographic, clinical, and prostate morphological characteristics were reviewed across IPP grades. Apart from expected differences in prostate morphology, no relevant baseline imbalances were observed across groups.

**Table 1:** Baseline demographic, clinical, and prostate-related characteristics of the study population

Variable	Value
Age (years)	61 (56–66)
BMI (kg/m²)	26.4 (25.0–28.3)
PSA (ng/mL)	1.3 (0.8–2.75)
*Comorbidities, n (%)*	
Hypertension	56 (26.1%)
Coronary artery disease	12 (5.6%)
Hyperlipidemia	3 (1.4%)
COPD	10 (4.6%)
*Prostate-related variables*	
Width-D1 (cm)	4.44 (4.04–4.89)
Height-D2 (cm)	4.36 (3.93–4.87)
Length-D3 (cm)	4.45 (3.96–5.29)
Prostate volume (mL)	45.0 (34.0–63.5)
*Grading of IPP (mm), n (%)*	
Grade I (< 5 mm)	73 (34.1%)
Grade II (5–10 mm)	78 (36.4%)
Grade III (> 10 mm)	63 (29.4%)

*BMI* body mass index,* PSA* prostate-specific antigen, *COPD* chronic obstructive pulmonary disease,* IPP* intravesical prostatic protrusion

Values are presented as median (interquartile range) or number (%)

The effects of tamsulosin treatment on urinary and ejaculatory parameters are summarized in Table 2. Following 8 weeks of therapy, the median IPSS significantly decreased from 18 (IQR, 16–22) to 14 (IQR, 12–17) (*p* < 0.001), while the median Qmax increased from 9.2 mL/s (IQR, 6.8–11.6) to 14.0 mL/s (IQR, 11.1–17.0) (*p* < 0.001).

Regarding ejaculatory outcomes, the median MSHQ-EjD-SF Q1–Q3 composite score declined from 11 (IQR, 10–12) to 7 (IQR, 6–8) (*p* < 0.001), indicating reduced ejaculatory function, whereas the bother score (Q4) increased from 2 (IQR, 1–2) to 4 (IQR, 3–5) (*p* < 0.001) (Table 2).

**Table 2:** Pre- and post-treatment changes in IPSS, Qmax, and MSHQ-EjD scores after Tamsulosin therapy

Variable	Pre-treatment	Post-Treatment	Δ (change)	*p* value
IPSS	18 (16–22)	14 (12–17)	↓ 4	*< 0.001* ^a^
Qmax (mL/s)	9.2 (6.8–11.6)	14.0 (11.1–17.0)	↑ 4.8	*< 0.001* ^a^
MSHQ-EjD-SF Q1-Q3 (max 15)	11 (10–12)	7 (6–8)	↓ 4	*< 0.001* ^a^
MSHQ-EjD-SF Q4	2 (1–2)	4 (3–5)	↑ 2	*< 0.001* ^a^

Values are presented as median (interquartile range). Δ indicates direction of change.

^a^Wilcoxon signed-rank test

Correlation analysis demonstrated that the change in ejaculatory function (ΔMSHQ-EjD) was negatively correlated with IPP (ρ = −0.386, *p* < 0.01), prostate length (ρ = −0.250, *p* < 0.01), and age (ρ = −0.334, *p* < 0.01), indicating that patients with larger IPP, longer prostates, or older age experienced less decline in ejaculatory function following tamsulosin therapy.

In contrast, ΔMSHQ-EjD was positively correlated with ΔIPSS (ρ = +0.318, *p* < 0.01) and negatively correlated with ΔQmax (ρ = −0.332, *p* < 0.01), suggesting that greater improvements in IPSS or Qmax were associated with more pronounced declines in ejaculatory function. No significant correlations were observed with prostate volume, width, or height (*p* > 0.05) (Table 3).

**Table 3:** Correlation between ΔMSHQ-EjD and clinical/anatomical parameters

Variable	ρ	*p* value
Age (years)	−0.334	< 0.01
Prostate volume (mL)	−0.120	0.08
Width-D1 (cm)	+ 0.035	0.61
Height-D2 (cm)	−0.027	0.69
Length-D3 (cm)	−0.250	< 0.01
IPP (mm)	−0.386	< 0.01
ΔIPSS	+ 0.318	< 0.01
ΔQmax	−0.332	< 0.01

ΔMSHQ-EjD: change in ejaculatory function score after tamsulosin therapy; IPP: intravesical prostatic protrusion; ΔIPSS: change in International Prostate Symptom Score;.ρ: Spearman’s correlation coefficients

**Fig. 2:**
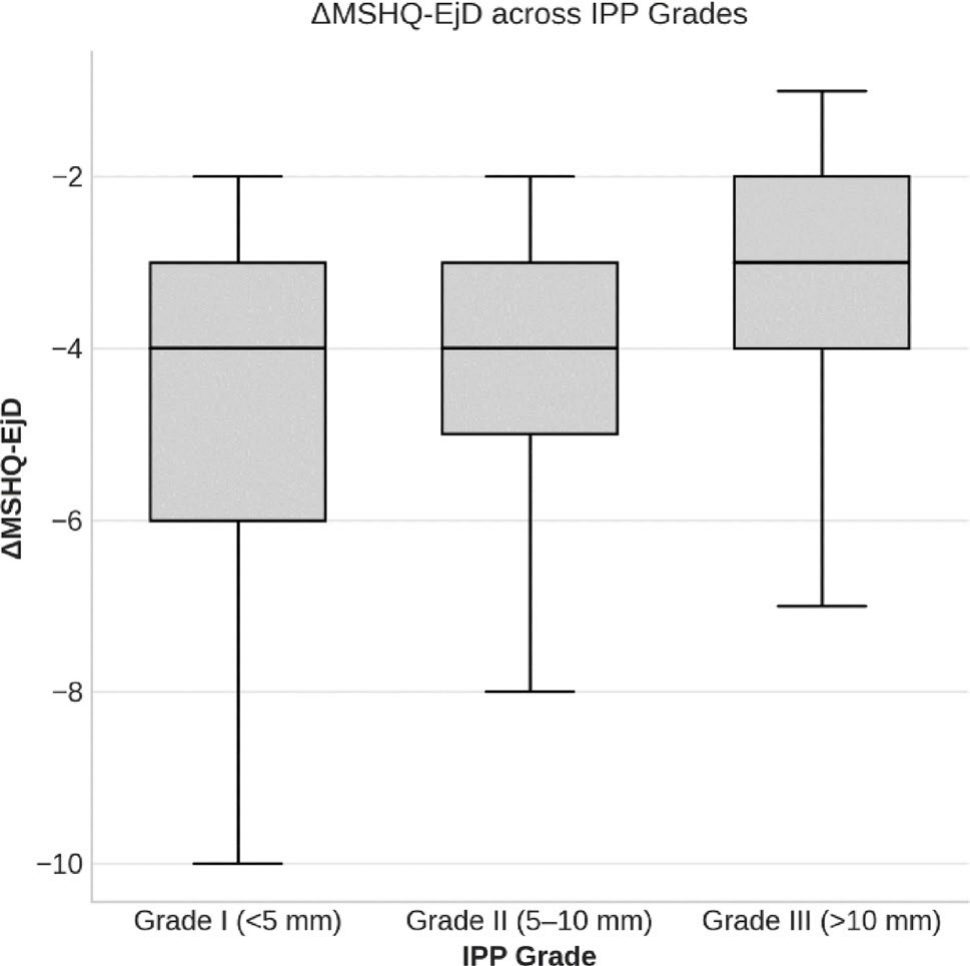
Distribution of ΔMSHQ-EjD scores across intravesical prostatic protrusion (IPP) grades, showing smaller declines in ejaculatory function with higher IPP grades

Comparison of ΔMSHQ-EjD across IPP grades revealed a significant overall difference (χ² = 35.4, *p* < 0.001, ε² = 0.166). Post-hoc analysis showed that patients with Grade III IPP (> 10 mm) had significantly higher (less negative) ΔMSHQ-EjD scores than those with Grade I (*p* < 0.001) and Grade II (*p* < 0.001), whereas no significant difference was found between Grade I and Grade II groups (*p* > 0.05). These findings indicate that patients with more prominent IPP experienced a relatively smaller decline in ejaculatory function following tamsulosin therapy (Table 4; Fig. 2).

**Table 4:** Comparison of ΔMSHQ-EjD across IPP grades

IPP Grade	*n*	ΔMSHQ-EjD	*p* value
Grade I (< 5 mm)	73	−4 (− 6 to − 3)	< 0.001 ^a^
Grade II (5–10 mm)	78	−4 (− 5 to − 3)	
Grade III (> 10 mm)	63	−3 (− 4 to − 2)	

Values are presented as median (interquartile range)

^a^ Kruskal-Wallis Test

## Discussion

In this prospective study, we investigated the relationship between changes in ejaculatory function following tamsulosin therapy and prostate morphology, particularly the degree of intravesical prostatic protrusion. Our findings demonstrated that, among patients receiving tamsulosin, higher IPP grades and greater craniocaudal prostate length were statistically associated with a less pronounced decline in ejaculatory function. Moreover, although significant improvements in IPSS and Qmax were achieved after tamsulosin treatment, these symptomatic benefits were accompanied by a statistically significant decline in ejaculatory function. This decline tended to be more pronounced among younger patients.

Ejaculation has long been the subject of extensive investigation and is widely recognized as a complex, multistep process involving coordinated anatomical and neurophysiological mechanisms. In this context, our results suggest that prostate morphology may be associated with differences in the ejaculatory response to tamsulosin therapy. To the best of our knowledge, no previous study has directly examined the relationship between prostate dimensions, including IPP, and tamsulosin-induced changes in ejaculatory function. In our cohort, patients with higher IPP grades and greater craniocaudal prostate length experienced a less pronounced decline in ejaculatory function, suggesting that certain anatomical configurations may modulate the functional impact of α1A-adrenergic blockade. Traditionally, bladder neck closure has been regarded as one of the components involved in antegrade ejaculation [16–18]. However, accumulating evidence from recent functional and surgical studies indicates that its role is likely secondary rather than central. Contemporary concepts increasingly emphasize the peri-verumontanum region, the prostatic apex, and the so-called ejaculatory hood as the primary functional fulcrum of ejaculatory control [19]. In this regard, the EJAC study by Pradere et al., conducted in young, healthy volunteers without lower urinary tract symptoms, demonstrated that antegrade ejaculation is predominantly governed by coordinated peri-verumontanum and apical prostatic activity rather than by bladder neck closure alone [20]. Similarly, the contemporary review by Sibona et al. highlighted the pivotal role of ejaculatory hood preservation in maintaining antegrade ejaculation [21]. However, the variability in antegrade ejaculation rates reported among ejaculation-preserving TURP (Transurethral Resection of the Prostate) and HoLEP (Holmium Laser Enucleation of the Prostate) techniques, despite consistent preservation of the peri-verumontanum region, suggests that additional anatomical factors, including bladder neck configuration, may also influence ejaculatory outcomes [22–25]. Within this updated conceptual framework, IPP should not be interpreted as a direct determinant of ejaculatory hood function. Rather, IPP and prostate length may reflect aspects of global prostate geometry that shape the anatomical and functional environment in which peri-verumontanum and apical structures operate. A contributory role of IPP-related bladder outlet configuration in facilitating bladder neck closure during ejaculation cannot be entirely excluded; however, if present, such an effect is likely secondary and context-dependent. Accordingly, the relatively smaller decline in ejaculatory function observed in patients with higher IPP and longer prostate length in our study may indicate that the disruptive effects of tamsulosin on ejaculatory coordination are less pronounced in certain anatomical configurations. These observations are correlational in nature and should be regarded as hypothesis-generating rather than indicative of a causal mechanism. Future studies incorporating dynamic endoscopic or imaging-based assessments of ejaculatory events in symptomatic BPH populations may help further elucidate these proposed mechanisms.

The association observed in our study between symptomatic improvement and ejaculatory decline is consistent with previous literature. In a meta-analysis by Gacci et al., ejaculatory dysfunction was positively correlated with improvements in IPSS and Qmax, and occurred more frequently in patients demonstrating a better therapeutic response [11]. Similarly, Song et al. reported greater reductions in MSHQ-EjD scores among patients with more pronounced IPSS improvement [12].

The correlation observed in our study between MSHQ-EjD score reduction and improvements in IPSS and Qmax supports these earlier findings. Collectively, these results are in line with the concept that α1A-selective agents such as tamsulosin, while effectively relaxing the smooth muscle of the lower urinary tract, may also attenuate sympathetic activity during the ejaculatory phase, thereby contributing to functional impairment [15]. However, it should be acknowledged that although these associations are statistically significant, the magnitude of ejaculatory score changes remains modest.

Age has also been identified as an important determinant of changes in ejaculatory function among patients treated with alpha-blockers [26, 27]. The more pronounced reduction in MSHQ-EjD scores observed in younger patients in our study parallels the age-related pattern reported by Chapple et al. [28]. The greater sympathetic tone and ejaculatory reflex activity in younger men may contribute to a more noticeable impact of α1A-receptor blockade induced by tamsulosin. Conversely, the physiological reduction in sympathetic activity observed with aging may be associated with a less pronounced effect on ejaculatory function [29].

Apart from these factors, several clinical studies have also evaluated tamsulosin-associated ejaculatory dysfunction. Stojanović et al. demonstrated a progressive decline in MSHQ-EjD scores among patients treated with tamsulosin 0.4 mg/day over 3 and 6 months (10.49 ± 2.43 vs. 7.46 ± 2.67 vs. 6.22 ± 2.31; *p* < 0.05) [30]. Furthermore, studies comparing different tamsulosin dosages have addressed the impact of dosing on ejaculatory function [31, 32]. In a phase III trial by Narayan et al., the incidence of abnormal ejaculation was higher in the 0.8 mg group compared with the placebo and 0.4 mg groups (18% vs. 11%; *p* ≤ 0.05) [33]. In our cohort, patients receiving 0.4 mg tamsulosin exhibited a significant reduction in MSHQ-EjD Q1–Q3 scores at the eighth-week follow-up compared with baseline (11 [10–12] vs. 7 [6–8]; *p* < 0.001). This change was accompanied by higher MSHQ-EjD Q4 (bother) scores (2 [1–2] vs. 4 [3–5]), reflecting greater ejaculatory bother.

This study has several limitations. Its single-center design and relatively small sample size limit the generalizability of the findings. Serum hormone levels, ejaculate volume, and seminal parameters were not assessed. The follow-up period of eight weeks was relatively short, preventing evaluation of long-term changes in ejaculatory function. Moreover, all prostate measurements were performed using transabdominal USG , which is inherently operator-dependent and may introduce variability. In addition, patients who discontinued tamsulosin due to side effects were excluded from the analysis, which may have introduced attrition bias. Furthermore, no multivariable analysis was performed; therefore, residual confounding between age, IPP, prostate length, and symptom severity cannot be excluded. Future long-term prospective studies with larger cohorts and comprehensive multivariable designs are warranted to address these methodological limitations.

## Conclusion

The present study indicates that prostate morphological characteristics—particularly IPP > 10 mm and longer prostate length—may be associated with a less pronounced decline in ejaculatory function during tamsulosin therapy. Although the observed correlations were modest, these findings provide a coherent framework for contextualizing the relationship between prostate anatomy and tamsulosin-related ejaculatory changes and support the need for confirmation in larger, multicenter, and longitudinal studies.

## Data Availability

No datasets were generated or analysed during the current study.
